# Antenatal maternal low protein diet: ACE-2 in the mouse lung and sexually dimorphic programming of hypertension

**DOI:** 10.1186/s12899-015-0016-6

**Published:** 2015-05-14

**Authors:** Ravi Goyal, Jonathan Van-Wickle, Dipali Goyal, Lawrence D. Longo

**Affiliations:** Center for Perinatal Biology, School of Medicine, Loma Linda University, Loma Linda, CA 92350 USA; Department of Basic Sciences, School of Medicine, Loma Linda University, Loma Linda, CA 92350 USA

**Keywords:** Angiotensin converting enzyme, microRNA, Epigenetics, DOHaD, Fetal programming, Developmental origins, Barker hypothesis

## Abstract

Elevated blood pressure is an important global health problem, and *in-utero* under-nutrition may be an important factor in the pathogenesis of hypertension. In the present study, we tested the hypothesis that antenatal maternal low protein diet (MLPD) leads to sexually dimorphic developmental programming of the components of the pulmonary renin-angiotensin system. This may be important in the antenatal MLPD-associated development of hypertension. In pregnant mice, we administered normal (control) and isocaloric 50 % protein restricted diet, commencing one week before mating and continuing until delivery of the pups. From the 18th to 24th week postnatal, we measured blood pressure in the offspring by use of a non-invasive tail-cuff method. In the same mice, we examined the mRNA and protein expression of the key components of the pulmonary renin-angiotensin system. Also, we examined microRNA complementary to angiotensin converting enzymes (ACE) 2 in the offspring lungs. Our results demonstrate that as a consequence of antenatal MLPD: 1) pup birthweight was significantly reduced in both sexes. 2) female offspring developed hypertension, but males did not. 3) In female offspring, ACE-2 protein expression was significantly reduced without any change in the mRNA levels. 4) miRNA 429, which has a binding site on ACE-2 - 3’ UTR was significantly upregulated in the female antenatal MLPD offspring. 5) In males, ACE-2 mRNA and protein expression were unaltered. We conclude that in the mouse, antenatal MLPD-induced reduction of ACE-2 in the female offspring lung may be an important mechanisms in sexually dimorphic programming of hypertension.

**Electronic supplementary material:**

The online version of this article (doi:10.1186/s12899-015-0016-6) contains supplementary material, which is available to authorized users.

## Background

More than 150 years after its first description, high blood pressure has been listed as the primary cause of death of 61,005 Americans in 2008 (www.heart.org/statistics). In 90 to 95% of these cases, the cause of hypertension is not known (AHA, 2008). Importantly, a family history of hypertension is an important risk factor for its development [1]; which suggests a strong genetic or familial environmental factor in its genesis. Moreover, during recent years, accumulating evidence indicates that to a significant degree, hypertension may have a developmental origin. Epidemiological data from human studies [2], and experiments in several species of laboratory animals including mice [3–6], rats [7], guinea pigs [8], and sheep [9], have demonstrated the importance of maternal nutrition during gestation in the genesis of hypertension in the adult offspring.

In humans, evidence supports sexually dimorphic trends in the occurrence of hypertension [10], with males being more affected than females before the latter’s menopause; and males and females being affected equally following female menopause [11]. Notably, with increasing age the blood pressure in females increases more rapidly than that of males [12]. The mechanisms of these changes are not known, however.

Following the discovery of renin as a pressor molecule [13], the renin-angiotensin system (RAS) has been established as one of the major pathways involved in both the development of hypertension and in fluid homeostasis. RAS has received increased attention, and our understanding of this system has changed radically over the past several decades. The cascade starts with an α_2_-globulin angiotensinogen (AGT), which is produced constitutively and released into the circulation, chiefly by the liver. A substrate for renin (secreted by kidneys), AGT is converted into the decapeptide angiotensin I (Ang I), and subsequently by angiotensin converting enzyme 1 (ACE 1) to the octapeptide Ang II. By the action of ACE-2, Ang II activity is terminated by its conversion to Ang 1–7. Of note, both ACE-1 and ACE-2 are secreted by the lung [14]. Importantly, a number of drugs (enalapril, lisinopril, etc.) used clinically [15–17] inhibit ACE1 and underscore the role of lungs in hypertension. Furthermore, studies have demonstrated that modulation of ACE from the lungs have important implications in the development of hypertension [18, 19]. Studies from our laboratory [3–5] and others [20] have demonstrated significant changes in the RAS in response to antenatal MLPD. However, the extent to which antenatal MLPD can lead to sexual dimorphic programing of the RAS in association with hypertension is not known. Also, the specific components of the RAS pathway that may be important in this programing are not well understood. Thus, we tested the hypothesis that antenatal MLPD leads to sexually dimorphic developmental programming of the components of the pulmonary renin-angiotensin system, which may be important in the antenatal MLPD-associated development of hypertension.

## Methods

### Experimental animal and tissues

The present study was in compliance with the Animal Welfare Act, guidelines of the American Physiological Society, and was approved by the Institutional Animal Care and Use Committee (IACUC) of Loma Linda University. We have described all these methods in our previous publications [3–6]. Briefly, we obtained FVB/NJ mice (~8 weeks of age) from Jackson Laboratories (Bar Harbor, ME), and housed them in the Animal Research Facility, Loma Linda University under conditions of 14 h light, 10 h darkness, ambient temperature of 20 °C, and relative humidity of 30 to 60 %. At 16 weeks of age, the mice were bred, by keeping the males and females together for 12 h (overnight). In the morning, mating was confirmed by examination of vaginal plugs, and considered 0.5 day post coitum (DPC). We started the study with 16 animals in each group. However, following overnight mating, the mice dams without vaginal plug and significant weight gain on day 7 post coitum were excluded from the study. This reduced the number of mice dams to 8 to 10 dams in each group. Following birth, 8 pups per group from 8 different mice dams were used to conduct the blood pressure measurement study. Molecular biology study (real-time PCR) were conducted on 4 animals from each group.

### Protein deprivation

Protein restricted chow was obtained from Newco Distributors Inc. (Rancho Cucamonga, CA). Mice dams were divided in two groups: control protein diet (18 g/100 g, 100 % protein content), and antenatal MLPD (9 g/100 g, 50 % protein diet). To avoid the stress of food change during gestation and to include the peri-conceptual period, diet administration was started one week before mating. To maintain an isocaloric diet with low protein, we replaced the proteins with carbohydrates. The normal diet contained 18 g protein/100 g food, as described (additional file 1) [3–6]. We measured the amount of food (g/day) consumed by the mice on both normal and isocaloric low protein diets; and these were similar, [being 3 ± 0.5 g for females (n = 16) and 4 ± 0.3 g for males (n = 16) in the control group and 3 ± 0.4 (n = 16) for females and 4 ± 0.3 g for males (n = 16) in the antenatal MLPD group, respectively].

### Physiological Measurement

We measured body weight on the morning after the pups were delivered (0.5 Days). Intact placentas were collected from the cage and placental weight was determined. After birth, all the mice dams were maintained on the control diet to examine the effect of antenatal developmental programming *per se*. Blood pressure was measured non-invasively, weekly by determining the tail blood volume, flow, and pressure with a volume pressure recording sensor and an occlusion tail-cuff (CODA System, Kent Scientific, Torrington, CT). This system is significantly different from the plethysmographic based tail-cuff measurement system, which measures only systolic blood pressure [21]. This is a highly accurate system with the capability of measuring systolic and diastolic blood pressures with the heart rate simultaneously and non-invasively [22, 23]. Moreover, in numerous other studies, the tail cuff method has been shown to measure the blood pressure non-invasively and accurately in mice and rats [24, 25]. Before commencing our studies, mice were placed on a warming plate at 37° centigrade until the temperature of the tail-region measured 37° by an infrared thermometer. Following the warming, the mice were trained for three 15-min sessions each day for three days, or until we obtained stable blood pressure recordings. Blood pressure was measured 20 times on a fixed day (Tuesday) and time (~11:30 AM), once weekly from 18 to 24 weeks of age.

### mRNA and protein quantification

At 32 weeks of age the mice were euthanized by cervical dislocation and the lungs were isolated. The isolated lungs were snap frozen in liquid nitrogen and stored at −80° centigrade for later analysis. For each experiment lung tissue from 4 different mice from different mothers were used. Real-time PCR and western immunoblot assays were conducted, as described and validated previously in our laboratory [3–5]. We isolated and quantified RNA and protein by Allprep DNA/RNA Mini Kit according to the manufacturer’s instructions (Qiagen Inc, Valencia, CA Cat # 80204). Isolated mRNA was analyzed using a NanoDrop1000 Spectrophotometer (Thermo Scientific, Waltham, MA) at 260/280 wavelength UV rays to check for quality and quantity. The 260/280 ratio of 1.8 to 2 was accepted for quantification with real-time PCR. Real-time PCR was performed on Light Cycler 1.5 (Roche Inc., Indianapolis, IN) using hydrolysis Taqman probes and primers, designed using the Universal Probe Library, a web based software (Roche Inc.), and the Quantfast Real-Time PCR Kit (Qiagen). Total RNA (1 ug per reaction) was reverse transcribed using Quantitect reverse transcriptase kit (Qiagen, Valencia, CA). Relative expression was normalized to 18S RNA and fold-changes were calculated using the ΔΔCt method with normalization of individual PCR efficiencies [26].

Western-immunoblot experiments were conducted as described previously [4, 5]. Briefly, frozen samples were homogenized in the 1 x cell lysing buffer (Cell Signaling Technology, Beverly, MA) containing a 1× phosphatase and protease inhibitor cocktail (Sigma). Nuclei and debris were pelleted by centrifugation at 1000 × g for 10 min. The supernatant was collected and stored at −80 °C. SDS-gel and western blot were performed by using appropriate antibodies (Table 1). All secondary antibodies were obtained from Abcam (Cambridge, MA). Twenty μg protein from each sample was loaded on a SDS-gel and electrophoresed at 100 V for 3 h. Proteins were transferred to a nitrocellulose membrane, and subjected to immunoblotting with antibodies. Bands were detected with enhanced chemiluminescence using a ChemiImager (Alpha-Innotech, San Leandro, CA). The results are expressed as fraction of control. We performed control experiments with glyceraldehyde-3-phosphate dehydrogenase (GAPDH), beta-tubulin, alpha-actin, and extracellular regulated signal kinase1/2 (ERK1/2). Our results demonstrated total ERK1/2 protein expression to be the most uniform among all the four study groups. Moreover, ERK1/2 integrated density (arbitrary unit) on densitometry analysis correlated well with different amounts of protein loaded, and was used as an internal control to account for uniform protein loading.

**Table 1:** Antibodies used in the study

No.	Name	Company	Cat. No.	Dilution
1	Angiotensinogen	Swant Inc. Switzerland	138	1:1000
2	Renin	Santa Cruz Biotechnologies	sc-27320	1:300
3	ACE-1	Abcam Inc.	ab39172	1:1000
4	ACE-2	Abcam Inc.	ab59351	1:1000
5	AT-1	Abcam Inc.	ab59018	1:250
6	AT-2	Abcam Inc.	ab19134	1:250
7	GAPDH	Abcam Inc.	ab9485	1:2000
8	Alpha-Actin	Abcam Inc.	Ab1801	1:1000
9	ERK1/2	Cell Signaling Technology	#9102	1:1000
10	Beta-tubulin	Santa Cruz Biotechnology	sc-5274	1:1000

### MicroRNA studies

MicroRNAs (miRNA) were identified for the 3’ UTR of the ACE-2 mRNAs, using the web based bio-informatics software – TargetScan 4.2 (http://www.targetscan.org). Of 10 miRNA suggested by the bio-informatics software, we chose mmu-mir-429 miRNA for the present study, by the use of the Context Score of more than 90^th^ percentile, as described by Grimson *et al.* [27]. The identified miRNA levels were measured by the use of Real-Time Taqman microRNA PCR assays, according to manufacturer’s instructions (Life Technologies, Grand Island, NY).

### Statistical analysis

We analyzed the data using repeated measure (RM) two-way Univariate Analysis of Variance (ANOVA) with Bonferroni’s and Tukey’s post-hoc analysis as well as linear and non-linear regression to determine statistically significant differences between groups, by the use of GraphPad Prism software (GraphPad Software Inc., San Diego, CA) and IBM SPSS (IBM Corp., NY). The hypothesis was accepted at P < 0.05. For the measurement of blood pressure one male or female offspring was included from one mice dam and considered n = 1.

## Results

### Offspring mice weight in response to antenatal protein restriction

**Fig. 1:**
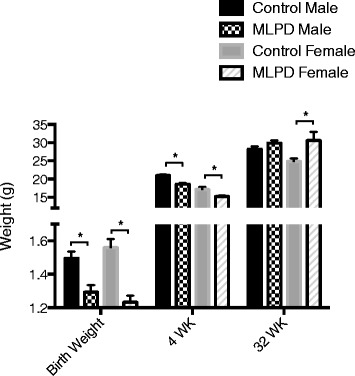
Weight in grams (g) of mice offspring at birth, 4 weeks, and 32 weeks of age from control and antenatal MLPD group. *Denotes P < 0.05. N = 16 for each group. MLPD - Mice offspring from the maternal low protein diet group

As a consequence of antenatal protein restriction during the entire gestation, we observed a significant reduction in birth weight in mice offspring. Both male and female antenatal MLPD were associated with a significant reduction in the birth weight (Fig. 1A), being (g) for control male 1.5 ± 0.04 (n = 8), antenatal MLPD male 1.3 ± 0.04 (n = 11), control female 1.6 ± 0.05 (n = 8), and antenatal MLPD female 1.2 ± 0.04 (n = 11). Of importance, the antenatal MLPD offspring demonstrated catch-up growth, so that by 32 weeks of age, female from the antenatal MLPD group were significantly over-weight. Males were affected to a significantly lesser degree, however (Fig. 1).

### Litter size and placental weight

Neither litter size nor placental weights differ significantly. The average litter size was 7 ± 2 and 6 ± 3 in the control and antenatal MLPD group, respectively (P > 0.05). The pups sex distribution was between 40 to 60 % per mice dam between control and MLPD group (P > 0.05).

### Blood pressure

**Fig. 2:**
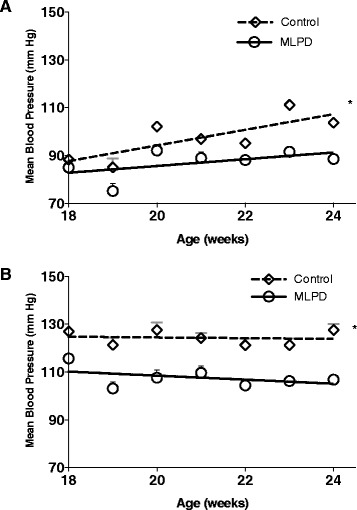
Mean arterial blood pressure for 7 weeks (18 weeks to 24 weeks of age) of male mice offspring **(A)** and female mice offspring **(B)** from control and antenatal MLPD group. *Denotes P < 0.05. N = 8 for each group. MLPD - Mice offspring from the maternal low protein diet group

We measured mean arterial blood pressure from the 18^th^ week to 24^th^ postnatal week (n = 8 mice in each group). “A sexually dimorphic trend of hypertension was observed in males and females as observed by a significant interaction of Sex*Diet by two-way RM ANOVA”. As shown in Fig. 2, the data demonstrates that antenatal MLPD female offspring had significantly higher mean arterial blood pressure, compared to the offspring from dams on the normal diet. In males, however, there was a significantly less increase in mean arterial blood pressure in response to antenatal MLPD, compared to females (Fig. 2).

### Renin angiotensin system expression

**Fig. 3:**
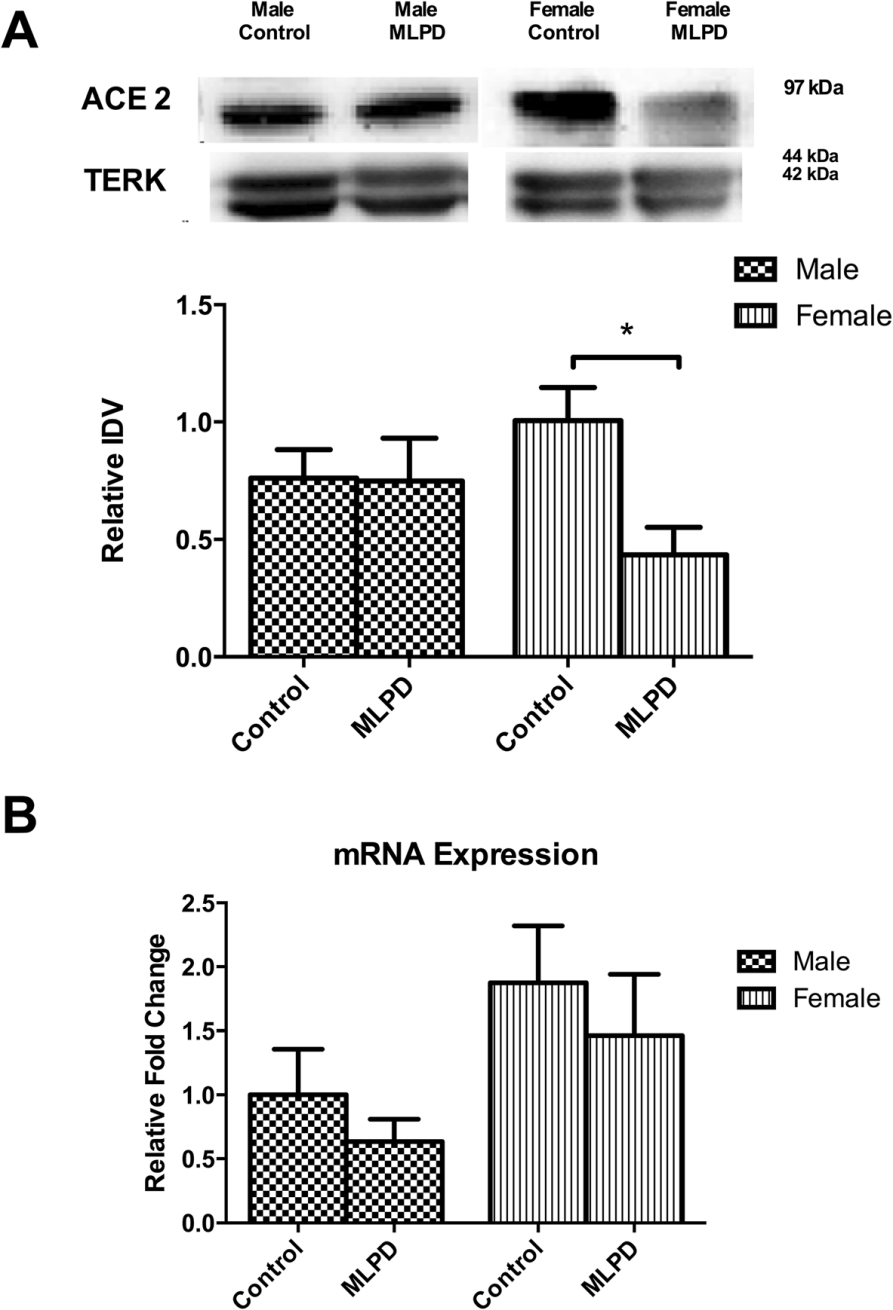
Expression levels of ACE2. **(A)** Western blot analysis **(B)** mRNA analysis of mice offspring from control and antenatal MLPD groups. IDV - integrated density value, an arbitrary unit. *Denotes P < 0.05. N = 4 for each group. MLPD - Mice Offspring from the maternal low protein diet group

To examine the underlying molecular alterations in association with hypertension, we examined several key components of the pulmonary renin-angiotensin system. With antenatal MLPD, we observed no significant changes in the protein expression of renin, ACE1, or angiotensin II type 1 and 2 receptors protein expression (Table 2). Notably, as shown in the Fig. 3A, in female antenatal MLPD offspring, ACE-2 protein expression was significantly reduced; whereas, no change was observed in the male offspring. To examine, the extent to which the alteration in the ACE-2 expression occurs at the transcriptional level, we conducted real-time PCR analysis of ACE-2 mRNA (Fig. 3B). No significant changes were observed in ACE-2 mRNA levels in antenatal MLPD lungs from either males or females.

**Table 2:** Protein levels of un-altered components of the pulmonary renin-angiotensin system in response to antenatal maternal low-protein diet

Gene Name	Males		Females	
	Protein Levels		Protein Levels	
	Control	MLPD	Control	MLPD
Renin	0.82 ± 0.07	0.92 ± 0.05	0.7 ± 0.1	0.7 ± 0.15
ACE1	0.94 ± 0.04	0.81 ± 0.03	0.28 ± 0.15	0.16 ± 0.01
AT1	0.15 ± 0.01	0.14 ± 0.03	0.19 ± 0.01	0.21 ± 0.03
AT2	0.08 ± 0.01	0.08 ± 0.02	0.1 ± 0.2	0.9 ± 0.02

Protein levels are shown as integrated density (arbitrary measurement) relative to total-ERK. N = 4, no statistically significant difference (P < 0.05) was identified by one way ANOVA and post-hoc Bonferroni’s in any of the measurement.

### ACE-2 complementary miRNA expression

**Fig. 4:**
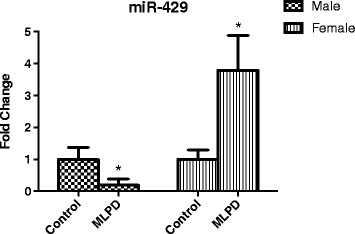
Expression levels of miR-429 from control and antenatal MLPD groups. *Denotes P < 0.05. N = 4 for each group. MLPD - Mice Offspring from the maternal low protein diet group

To examine, whether the reduction in ACE-2 protein levels in the female antenatal MLPD group is as a consequence of reduced translation, we examined miRNA miR-429, which has a complementary binding site on 3’ UTR of ACE-2. Of importance, as shown in Fig. 4 miR-429 levels were significantly increased in the female and decreased in maleantenatal MLPD lungs, compared to controls.

## Discussion

Accumulating evidence suggests that antenatal maternal protein deprivation can lead to the development of hypertension in the offspring [6, 7, 9, 28, 29]. Of note, in a manner similar to humans, mice also show an increase in blood pressure with aging [30]. The mechanisms of this phenomenon are not known, however. In the present study, we identified that in females, antenatal MLPD-induced hypertension is associated with a reduction in the level of ACE-2 enzyme (Fig. 3). Importantly, female mice offspring were affected with hypertension to a significantly greater extent than males (Fig. 2), which may relate to the ACE-2 gene being located on the X chromosome [31]. Further, studies in three different rat strains have demonstrated that reduced ACE-2 expression is associated with hypertension [32]. In addition, ACE-2 gene maps to a defined quantitative trait locus (QTL) for increased blood pressure on the X chromosome in these hypertensive rats [32].

Several other reports also have suggested an important role of ACE-2 in hypertension [33, 34]. In the present study, as a consequence of antenatal MLPD, we observed that ACE-2 was reduced only in female, and not male offspring (Fig. 3A). Importantly, only female offspring from the antenatal MLPD group were significantly hypertensive (Fig. 2). It appears that, as a consequence of antenatal MLPD, in females ACE-2 is differentially regulated, and may play an important role in the developmental programming of hypertension. Being located on an X chromosome, ACE-2 gene is susceptible for sexually dimorphic programming, as the chromosome is well known for other sexually dimorphic gene expression and disorders [35]. Additionally, ACE-2 is known to be regulated in a sexually dimorphic manner by estrogen [36]. Also, a high fat diet in rats is known to cause sexually dimorphic regulation of ACE-2 and the development of hypertension [37]. The present study, adds to the accumulating evidence that ACE-2 can be developmentally programmed by antenatal stressors during gestation, and may be a critical factor in the development of hypertension.

**Fig. 5:**
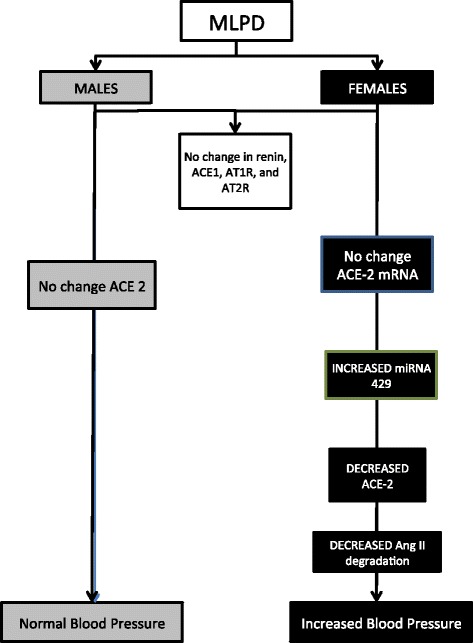
Summarized findings of the study

Of vital importance, in the present study, we identified that in response to antenatal MLPD, ACE-2 is not regulated at the transcriptional level, but rather programming occurs at the translational level. The ACE-2 mRNA levels were not significantly altered as a consequence of antenatal maternal protein deprivation (Fig. 3B); however, the ACE-2 protein levels were significantly lower in the antenatal MLPD females. Recent studies have demonstrated that miRNA are important in post-translational gene regulation, and play an important role in the developmental programming of hypertension. miRNA can bind to the 3’ UTR of a near-complementary sequence of mRNA and lead to reduced translation. As noted in Fig. 4, we observed that miRNA 429, which is complementary to ACE-2 was significantly upregulated, which agrees with other studies [14, 38, 39]. Importantly, there was no increase in ACE2 proteins and associated miRNA-429 in lungs from the male MLPD group. As shown in the summary Fig. 5 and previous studies, we speculate that antenatal maternal protein restriction leads to increased miR-429, which causes reduced production of ACE-2 protein. Reduced levels of ACE-2 lead to reduced degradation of the potent vasoconstrictor angiotensin II and thus increased blood pressure.

## Conclusion

Developmental programming of adult health and disease is an important factor in the genesis of hypertension. The present study, demonstrates that sexual dimorphism of the development of hypertension may be mediated through miRNA-mediated mechanisms and alterations in the ACE-2 gene. The questions remain, by what mechanism miRNA expression is regulated and programmed by in-utero environment? What is the role of estrogen in this signaling? We shall pursue thesein future studies.

## Additional files

Additional file 1: Table S1. Composition of the diet administered to control group of mice dams. Table S2 Composition of the diet administered to the antenatal maternal low protein group of mice dams.
