# Supplementary material for: Antenatal maternal low protein diet: ACE-2 in the mouse lung and sexually dimorphic programming of hypertension
Source: BMC Physiol. 2015 May 14;15:2. doi: 10.1186/s12899-015-0016-6 (PMC4430899; doi:10.1186/s12899-015-0016-6)
Supplement: Additional file 1: Table S1. — Composition of the diet administered to control group of mice dams. Table S2 Composition of the diet administered to the antenatal maternal low protein group of mice dams. [file 12899_2015_16_MOESM1_ESM.pdf]

Supplemental Table 1

Normal Diet - 10% Fat & 18% Protein

NUTRITIONAL PROFILE [1]

Protein, %	***18.0***
Arginine, %	0.69
Histidine, %	0.50
Isoleucine, %	0.94
Leucine, %	1.69
Lysine, %	1.42
Methionine, %	1.00
Cystine, %	0.07
Phenylalanine, %	0.94
Tyrosine, %	0.99
Threonine, %	0.76
Tryptophan, %	0.22
Valine, %	1.12
Alanine, %	0.54
Aspartic Acid, %	1.26
Glutamic Acid, %	4.00
Glycine, %	0.38
Proline, %	2.31
Serine, %	1.08
Taurine, %	0.00
Fat, %	***10.1***
Cholesterol, ppm	0
Linoleic Acid, %	5.72
Linolenic Acid, %	0.09
Arachidonic Acid, %	0.00
Omega-3 Fatty Acids, %	0.09
Total Saturated Fatty A	1.27
Total Monounsaturated Fatty Acids, %	2.42
Polyunsaturated Fatty Acids, %	5.81
Fiber (max), %	***3.8***
Carbohydrates, %	***62.8***
Energy (kcal/g) [2]	***4.11***

From:	kcal	%
Protein	0.720	17.4
Fat (ether extract)	0.907	21.9
Carbohydrates	2.513	60.7

Minerals	
Calcium, %	0.52
Phosphorus, %	0.56
Phosphorus (available), %	0.56
Potassium, %	0.36
Magnesium, %	0.05
Sodium, %	0.12
Chloride, %	0.21
Fluorine, ppm	0.0
Iron, ppm	35
Zinc, ppm	35
Manganese, ppm	59
Copper, ppm	6.0
Cobalt, ppm	0.0
Iodine, ppm	0.21
Chromium, ppm	2.0
Molybdenum, ppm	0.00
Selenium, ppm	0.11
Vitamins	
Vitamin A, IU/g	4.0
Vitamin D-3 (added), IU/g	1.0
Vitamin E, IU/kg	50.0
Vitamin K (as menadione), ppm	0.50
Thiamin Hydrochloride, ppm	6.1
Riboflavin, ppm	6.7
Niacin, ppm	30
Pantothenic Acid, ppm	16
Folic Acid, ppm	2.1
Pyridoxine, ppm	5.8
Biotin, ppm	0.2
Vitamin B-12, mcg/kg	14
Choline Chloride, ppm	1,000
Ascorbic Acid, ppm	0.0

1. Based on the latest ingredient analysis information. Since nutrient composition of natural ingredients varies, analysis will differ accordingly. Nutrients expressed as percent of ration on an As-Fed basis except where otherwise indicated.
2. Energy (kcal/gm) - Sum of decimal fractions of protein, fat and carbohydrate x 4,9,4 kcal/gm respectively.

Supplemental Table 2

Modest Protein Restriction Diet - 10% Fat & 9% Protein

NUTRITIONAL PROFILE [1]

Protein, %	***9.0***
Arginine, %	0.33
Histidine, %	0.24
Isoleucine, %	0.45
Leucine, %	0.80
Lysine, %	0.68
Methionine, %	0.74
Cystine, %	0.03
Phenylalanine, %	0.45
Tyrosine, %	0.47
Threonine, %	0.36
Tryptophan, %	0.10
Valine, %	0.53
Alanine, %	0.26
Aspartic Acid, %	0.60
Glutamic Acid, %	1.90
Glycine, %	0.18
Proline, %	1.10
Serine, %	0.51
Taurine, %	0.00
Fat, %	***10.1***
Cholesterol, ppm	0
Linoleic Acid, %	5.72
Linolenic Acid, %	0.09
Arachidonic Acid, %	0.00
Omega-3 Fatty Acids, %	0.09
Total Saturated Fatty A	1.27
Total Monounsaturated Fatty Acids, %	2.42
Polyunsaturated Fatty Acids, %	5.81
Fiber (max), %	***5.0***
Carbohydrates, %	***71.9***
Energy (kcal/g) [2]	***4.12***

From:	kcal	%
Protein	0.360	8.7
Fat (ether extract)	0.909	21.9
Carbohydrates	2.876	69.4

Minerals	
Calcium, %	0.52
Phosphorus, %	0.48
Phosphorus (available), %	0.47
Potassium, %	0.36
Magnesium, %	0.05
Sodium, %	0.13
Chloride, %	0.21
Fluorine, ppm	0.0
Iron, ppm	35
Zinc, ppm	32
Manganese, ppm	59
Copper, ppm	6.0
Cobalt, ppm	0.0
Iodine, ppm	0.21
Chromium, ppm	2.0
Molybdenum, ppm	0.00
Selenium, ppm	0.11
Vitamins	
Vitamin A, IU/g	4.0
Vitamin D-3 (added), IU/g	1.0
Vitamin E, IU/kg	50.0
Vitamin K (as menadione), ppm	0.50
Thiamin Hydrochloride, ppm	6.0
Riboflavin, ppm	6.3
Niacin, ppm	30
Pantothenic Acid, ppm	15
Folic Acid, ppm	2.1
Pyridoxine, ppm	5.8
Biotin, ppm	0.2
Vitamin B-12, mcg/kg	12
Choline Chloride, ppm	1,000
Ascorbic Acid, ppm	0.0

1. Based on the latest ingredient analysis information. Since nutrient composition of natural ingredients varies, analysis will differ accordingly. Nutrients expressed as percent of ration on an As-Fed basis except where otherwise indicated.
2. Energy (kcal/gm) - Sum of decimal fractions of protein, fat and carbohydrate x 4,9,4 kcal/gm respectively.
